# Chemoresponse of de novo Acute Myeloid Leukemia to “7+3” Induction can Be Predicted by c-Myc-facilitated Cytogenetics

**DOI:** 10.3389/fphar.2021.649267

**Published:** 2021-04-08

**Authors:** Tzu-Hung Hsiao, Ren Ching Wang, Tsai-Jung Lu, Chien-Hung Shih, Yu-Chen Su, Jia-Rong Tsai, Pei-Pei Jhan, Cai-Sian Lia, Han-Ni Chuang, Kuang-Hsi Chang, Chieh-Lin Teng

**Affiliations:** ^1^ Department of Medical Research, Taichung Veterans General Hospital, Taichung, Taiwan; ^2^ Department of Pathology, Taichung Veterans General Hospital, Taichung, Taiwan; ^3^ Department of Nursing, College of Nursing, Hungkuang University, Taichung, Taiwan; ^4^ Division of Hematology/Medical Oncology, Department of Medicine, Taichung Veterans General Hospital, Taichung, Taiwan; ^5^ Department of Medical Research, Tungs’ Taichung Metroharbor Hospital, Taichung, Taiwan; ^6^ Graduate Institute of Biomedical Sciences, China Medical University, Taichung, Taiwan; ^7^ General Education Center, Jen-The Junior College of Medicine, Nursing and Management, Miaoli, Taiwan; ^8^ Department of Life Science, Tunghai University, Taichung, Taiwan; ^9^ School of Medicine, Chung Shan Medical University, Taichung, Taiwan

**Keywords:** induction chemotherapy, “7+3”, myc (c-Myc), acute myeloid leukemia, chemoresponse

## Abstract

**Background:** Identifying patients with *de novo* acute myeloid leukemia (AML) who will probably respond to the “7 + 3” induction regimen remains an unsolved clinical challenge. This study aimed to identify whether c-Myc could facilitate cytogenetics to predict a “7 + 3” induction chemoresponse in *de novo* AML.

**Methods:** We stratified 75 untreated patients (24 and 51 from prospective and retrospective cohorts, respectively) with *de novo* AML who completed “7 + 3” induction into groups with and without complete remission (CR). We then compared Myc-associated molecular signatures between the groups in the prospective cohort after gene set enrichment analysis. The expression of c-Myc protein was assessed by immunohistochemical staining. We defined high c-Myc-immunopositivity as > 40% of bone marrow myeloblasts being c-Myc (+).

**Results:** Significantly more Myc gene expression was found in patients who did not achieve CR by “7 + 3” induction than those who did (2439.92 ± 1868.94 vs. 951.60 ± 780.68; *p* = 0.047). Expression of the Myc gene and c-Myc protein were positively correlated (r = 0.495; *p* = 0.014). Although the non-CR group did not express more c-Myc protein than the CR group (37.81 ± 25.13% vs. 29.04 ± 19.75%; *p* = 0.151), c-Myc-immunopositivity could be a surrogate to predict the “7 + 3” induction chemoresponse (specificity: 81.63%). More importantly, c-Myc-immunopositivity facilitated cytogenetics to predict a “7 + 3” induction chemoresponse by increasing specificity from 91.30 to 95.92%.

**Conclusion:** The “7 + 3” induction remains the standard of care for *de novo* AML patients, especially for those without a high c-Myc-immunopositivity and high-risk cytogenetics. However, different regimens might be considered for patients with high c-Myc-immunopositivity or high-risk cytogenetics.

## Introduction

With an incidence of 1.3 per 100,000 people, acute myeloid leukemia (AML) is the most common type of leukemia in adults (De Kouchkovsky and Abdul-Hay, 2016). Characterized by clonal expansion of immature myeloid blasts due to abnormal proliferation and differentiation of hematopoietic stem cells, ≥ 20% of nucleated cells from either peripheral blood or bone marrow being myeloblasts meet the World Health Organization AML diagnostic criteria (Vardiman et al., 2009). The current AML treatment flow includes the achievement of complete remission (CR) via induction chemotherapy followed by consolidation chemotherapy. Allogeneic hematopoietic stem cell transplantation (allo-HSCT) further improves the overall survival (OS) of patients with high-risk cytogenetics or genetic mutations, mainly when they are in CR (Burnett et al., 2011). Nevertheless, CR is the most crucial step toward curative intention in AML treatment.

Currently, the cytarabine 100 mg/m^2^ for 7 days; idarubicin 12 mg/m^2^ for 3 days (“7 + 3”) regimen is the standard of care among various types of induction regimens against AML. With relatively tolerable toxicities, this induction therapy can achieve a 70% CR rate in untreated *de novo* AML (Burnett et al., 2011). However, the outcomes for patients who do not achieve CR with “7 + 3” induction chemotherapy are exceptionally dismal. Several studies have focused on the possible mechanisms of induction failure in AML. From the perspective of cell functions, cell quiescence, DNA damage repair, and leukemic stem cell-related leukemogenesis might be associated with chemoresistance in AML (Abdullah and Chow, 2013; Vidal et al., 2014; Walter et al., 2015; Ng et al., 2016). In terms of clinical features, more advanced age, leukocytosis, and high-risk cytogenetics could be risk factors of “7 + 3” induction failure (Ho and Becker, 2013). However, the precise identification of patients with *de novo* AML who will probably respond to the “7 + 3” induction regimen remains an unresolved clinical challenge.

The Myc family consists of the nuclear transcription factors c-Myc, n-Myc, and i-Myc. Among the various Myc proteins, c-Myc plays a crucial role in most oncogenic processes, orchestrating proliferation, apoptosis, differentiation, and metabolism (Chen et al., 2018). Moreover, c-Myc is associated with chemoresistance in various cancers (Lee et al., 2017; Elbadawy et al., 2019). We previously revealed that Myc signature gene expression was higher in patients with *de novo* AML who failed to achieve CR by “7 + 3” induction than patients did (Chiu et al., 2019). Consequently, Myc could be a biomarker, facilitating chemoresistance prediction in *de novo* AML patients undergoing “7 + 3” induction therapy. However, studies of this clinical application remain limited. Therefore, we aimed to determine the value of Myc as part of a timely and practical approach to predict a chemoresponse to “7 + 3” induction.

The present study aimed to validate the role of Myc in chemoresistance to the “7 + 3” regimen in *de novo* AML. We also correlated expression of the Myc gene to that of c-Myc protein in 24 prospective patients with *de novo* AML who completed “7 + 3” or “7 + 3”-like induction chemotherapy. We then investigated whether c-Myc protein could facilitate cytogenetics to precisely predict a chemoresponse to “7 + 3” induction in a timely manner among patients with AML.

## Materials and Methods

### Patients

Between 2017 and 2020, we prospectively screened consecutive patients with untreated *de novo* non-promyelocytic AML (age ≤75 years; Eastern Cooperative Oncology Group Performance Status ≤2) who had completed the first cycle of cytarabine (100 mg/m^2^) for 7 days and idarubicin (12 mg/m^2^ for 3 days; “7 + 3”) or “7 + 3”-like induction chemotherapy. No other chemotherapeutic or novel agents were added to the “7 + 3” induction regimen regardless of the genetic mutation status of the patients. A total of 31 patients met these criteria. Seven patients were excluded from the study because no qualified RNA was extracted from the bone marrow leukemic cells for RNA sequencing at initial diagnosis. Finally, 24 patients were assigned to a prospective cohort (n = 24) and stratified into groups with (n = 15) and without (n = 9) CR according to their responses to the first cycle of “7 + 3” induction therapy.

To expand the number of study participants, we used data from a retrospective cohort comprising 52 patients with *de novo* AML who had completed “7 + 3” induction therapy (Chiu et al., 2019). One patient was excluded because of a disqualified bone marrow specimen that was ineligible for c-Myc immunohistochemical (IHC) staining. Finally, 51 patients were assigned to a retrospective cohort (n = 51). A combined cohort (n = 75) comprising prospective (n = 24) and retrospective (n = 51) patients was established for c-Myc-associated analyses. To avoid pathogenic background heterogeneity, this study did not include patients with therapy-related AML or AML with myelodysplasia-related changes. This study was approved by the Institutional Review Board of Taichung Veterans General Hospital and was in accordance with the Declaration of Helsinki (2013). All patients in the prospective cohort provided written informed consent to participate in the study before enrollment. The Institutional Review Board waived the need for informed consent for the retrospective cohort.

### RNA Sequencing

We prepared mononuclear cells from bone marrow aspirate specimens of the prospective cohort using BD Vacutainer^®^ CPT™ Mononuclear Cell Preparation Tube (Becton Dickinson and Co., Franklin Lakes, NJ, United States) as described by the manufacturer. Total RNA was extracted from mononuclear cells using TRIzol (Thermo Fisher Scientific Inc., Waltham, MA, United States) then purified using RNeasy Mini Kits and dnase I (Qiagen, Valencia, CA, United States). After enrichment using oligo (dT)-labelled magnetic beads, mRNA was fragmented, converted into cDNA, which was ligated to adaptors, and amplified. Quality-checked library products were 75-bp paired-end sequenced using a NextSeq 500 sequencer (Illumina Inc., San Diego, CA, United States). The original RNA-sequencing data from the prospective cohort has been deposited in the GEO repository (https://www.ncbi.nlm.nih.gov/geo/query/acc.cgi?acc=GSE164894).

After removing low-quality raw sequencing reads containing adaptor sequences or reads with high content of unknown bases, clean reads were aligned to the Ensembl GRCh38 human reference genome using HISAT2 (Kim et al., 2019). We used featureCounts software to count the mapped reads against Ensembl annotated genes (ENSG IDs) (Liao et al., 2014). Gene-level read counts were then normalized using DESeq2 and differential expression between AML patients with and without CR was assessed (Love et al., 2014).

### Myc Gene Set Enrichment Analysis and Myc Gene Quantitation

We identified Myc-associated molecular signatures curated in the Molecular Signatures Database (MSigDB) collections (Liberzon et al., 2011) using gene set enrichment analysis (GSEA) software. For each Myc-associated signature gene panel, GSEA reported leading-edge component genes, accounting for enrichment. We further compared Myc gene expression between the groups with and without CR in the prospective cohort using DESeq2 normalization.

### Immunohistochemical Staining for c-Myc Expression

Formalin-fixed paraffin-embedded tissue sections from bone marrow biopsies were stained for c-MYC (clone EP121, BioSB) on a Ventana BenchMark XT slide preparation system (Ventana Medical Systems, Tucson, AZ, United States). We decalcified bone marrow specimens with acid before October 2017. Thereafter, bone marrow biopsy samples were routinely decalcified with EDTA. An experienced hematological pathologist who was blinded to the genetic test results scored portions of c-Myc (+) myeloblasts from 0 to 100% to quantify c-Myc protein expression. We defined high c-Myc-immunopositivity when >40% of myeloblasts in the bone marrow were c-Myc (+) by testing the sensitivity and specificity according to different cutoffs from the combined cohort (Supplemental Table S1). We also examined c-Myc-immunopositivity in 20 normal bone marrow biopsy specimens to avoid interference by c-Myc overexpression in normal hematopoietic cells. All 20 bone marrow specimens contained <5% c-Myc (+) hematopoietic cells.

### Statistical Analysis

Continuous and categorical variables between the CR and non-CR groups were compared using Student t-tests and the Chi-squared tests, respectively. Numerical data are presented as means ± standard deviation. We applied logistic proportional regression to identify factors for high c-Myc-immunopositivity quantified according to odds ratios (OR) and 95% confidence intervals (CI). Values were considered statistically significant at *p* < 0.05.

## Results

### Patient Characteristics

Table 1 shows a demographic comparison between the groups with and without CR from the prospective cohort (n = 24). Sex (*p* = 0.669), age (*p* = 0.614), leukocyte count at diagnosis (*p* = 0.335), and cytogenetic risk (*p* = 0.057) did not significant differ between the groups. Proportions of FLT3 ITD (*p* = 0.615) and NPM1 (*p* = 0.615) gene mutations were also comparable between the groups. We also compared demographic data between the prospective and retrospective cohorts. These two cohorts had comparable demographic characteristics. This result revealed the absence of significant clinical heterogeneity between the prospective and retrospective cohorts (Supplemental Table S2).

**TABLE 1:** Characteristics of patients in the prospective cohort.

Variable	All patients (n = 24)	Non-CR group (n = 9)	CR group (n = 15)	p
Sex (n, %)				0.669^a^
Male	17 (70.83)	7 (77.78)	10 (66.67)	
Female	7 (29.17)	2 (22.22)	5 (33.33)	
Age, y (mean ± SD)	51.75 ± 13.57	49.89 ± 15.93	52.87 ± 12.42	0.614^b^
Leukocytes, 10^3^/μL (mean ± SD)	51.35 ± 51.08	64.47 ± 55.08	42.92 ± 48.51	0.335^b^
Cytogenetics (n, %)				0.057^a^
Favorable	3 (12.50)	0 (0)	3 (20.00)	
Intermediate	14 (58.33)	4 (44.44)	10 (66.67)	
Unfavorable	7 (29.17)	5 (55.56)	2 (13.33)	
Molecular risk (n, %)				
FLT3 ITD mutation	4 (16.67)	2 (22.22)	2 (13.33)	0.615^a^
NPM1 mutation	5 (20.83)	1 (11.11)	4 (26.67)	0.615^a^

^a^ Chi-squared.

^b^ *t*-tests. All data are shown as means ± SD or n (%).

CR, complete response; SD, standard deviation.

### Patients with AML Without CR Under “7 + 3” Induction Therapy had More Myc Gene Expression

**FIGURE 1:**
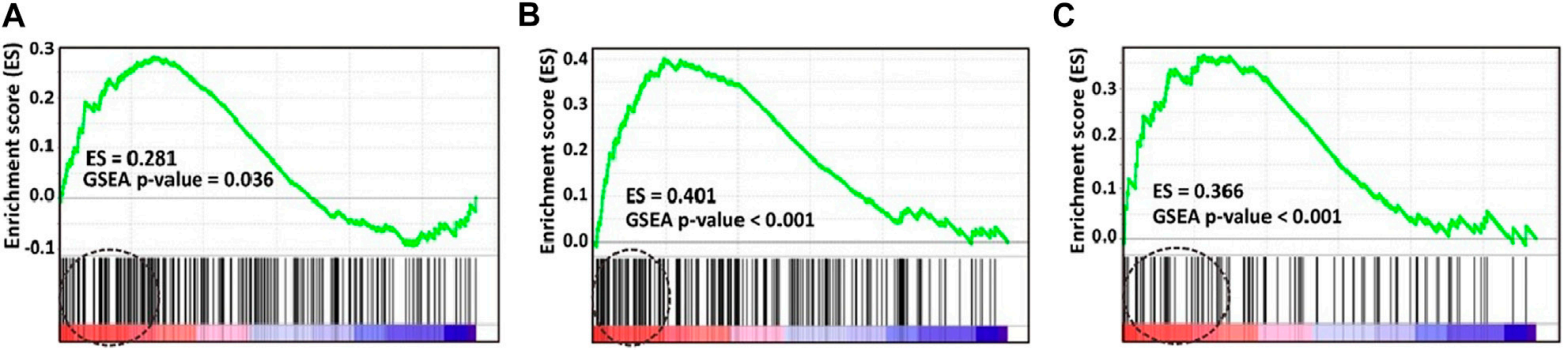
Gene set enrichment analysis (GSEA) plots of three selected molecular signatures for Myc. Enrichment score (ES) generated by Myc signatures were **(A)** 0.281 (p = 0.036) **(B)** 0.401 (p < 0.001), and **(C)** 0.366 (p < 0.001). Molecular signature gene expression of Myc is significantly higher in patients without, than with CR. CR, complete remission.

To validate our previous findings that Myc overexpression is associated with “7 + 3” induction chemoresistance in AML (Chiu et al., 2019), we compared Myc molecular signature gene expression between the groups with and without CR in the prospective cohort. Using three different Myc molecular signatures from MSigDB selected based on our best knowledge (Schlosser et al., 2005; Sansom et al., 2007), significantly more Myc molecular signature gene expression was found in the group that did not achieve CR than the group that did (Figure 1A,B,C).

**FIGURE 2:**
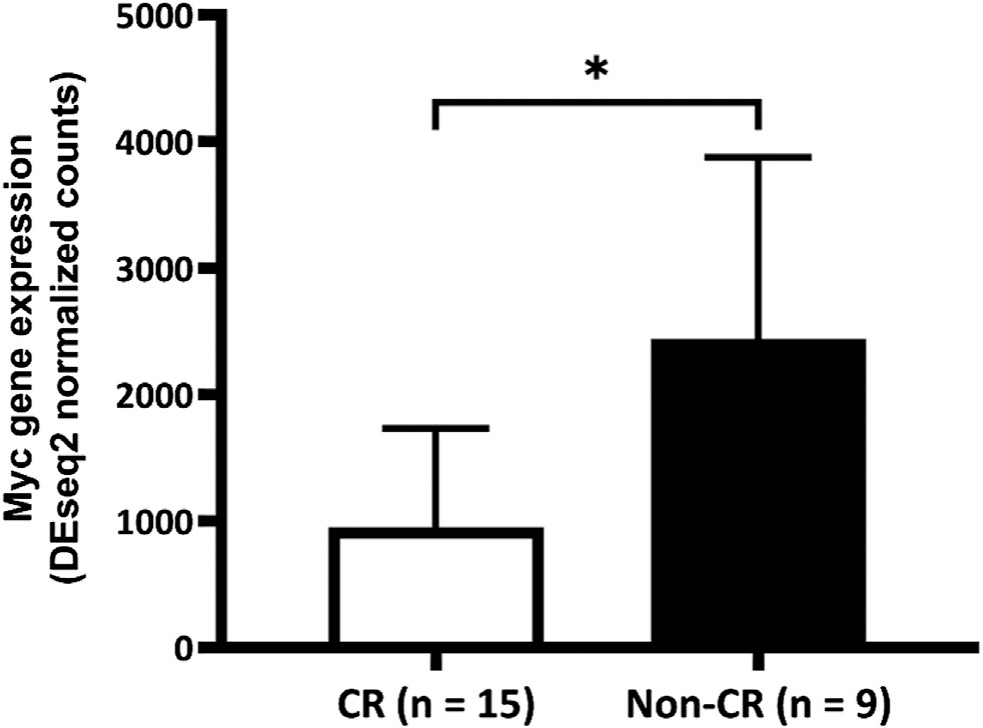
Comparison of Myc gene expression between the complete remission (CR) and non-CR groups. Expression of Myc gene is significantly higher in the group without than with CR (2439.92 ± 1868.94 vs. 951.60 ± 780.68, *p* = 0.047). CR, complete remission.

After confirming that patients with untreated *de novo* AML who did not achieve CR with the "7 + 3″ induction therapy expressed more of the Myc signature gene, we further investigated whether Myc gene expression differ between groups in the prospective cohort. The results showed that mean (±SD) amounts of Myc gene expression in the groups without and with CR were 2439.92 ± 1868.94 vs. 951.60 ± 780.68, *p* = 0.047; Figure 2).

### C-Myc Protein Expression Comparison Between the CR and non-CR Groups

**FIGURE 3:**
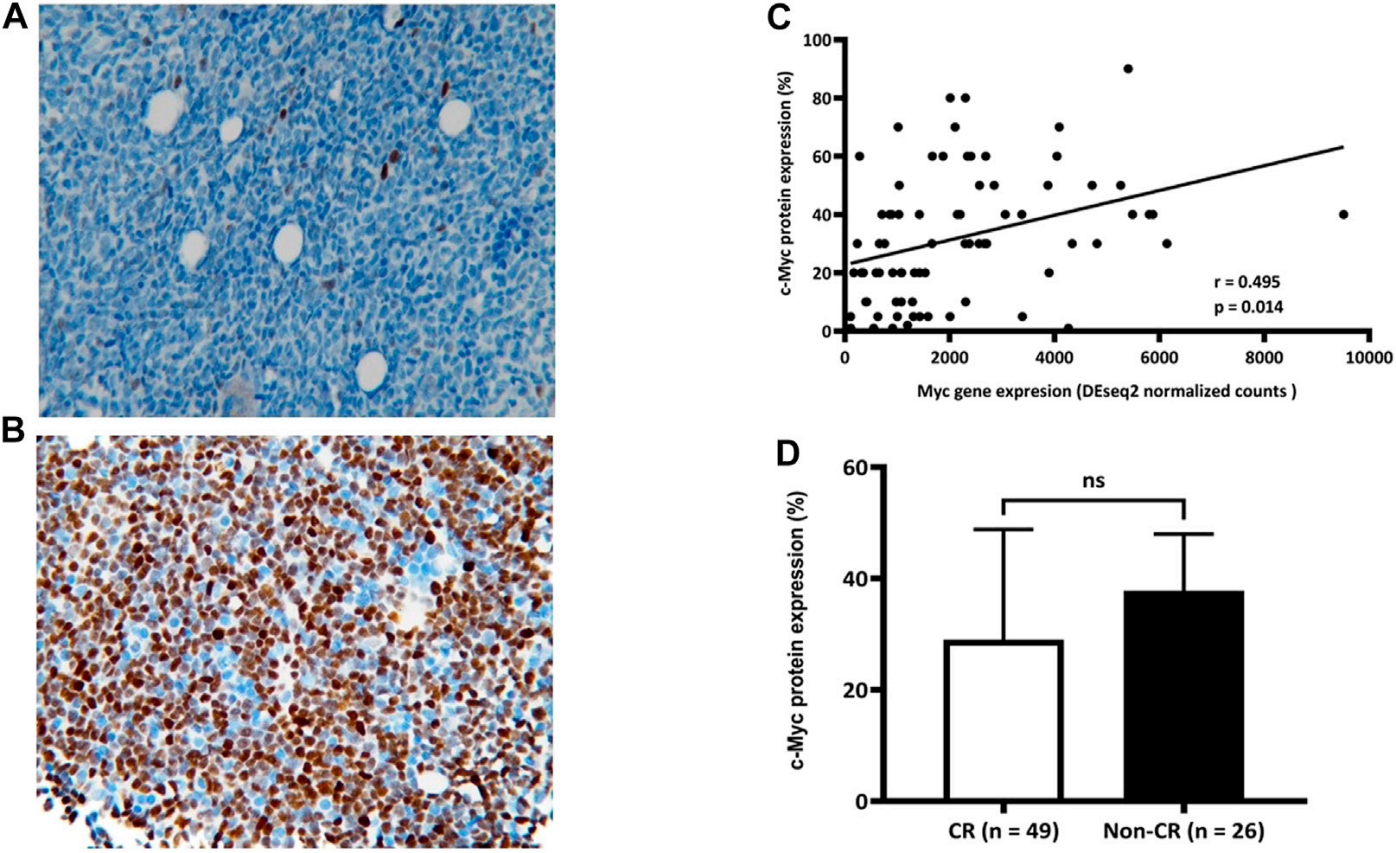
Correlation between expression of c-Myc protein and Myc gene. Average ratio of c-Myc (+) bone marrow myeloblasts in combined cohort is 32.08% (range, < 1% **(A)** to >90% **(B)**; n = 75). Significant correlation **(C)** between c-Myc protein and Myc gene expression (r = 0.495; *p* = 0.014) **(D)** Ratios of c-Myc (+) bone marrow myeloblasts between groups with and without CR (29.04 ± 19.75 and 37.81 ± 25.13, respectively; *p* = 0.151). CR, complete remission.

We aimed to develop a more timely and feasible approach to predict a chemoresponse of *de novo* AML to “7 + 3” based on our findings that patients with AML who did not achieve CR had more Myc gene expression. We assessed bone marrow myeloblasts for c-Myc protein by immunohistochemical (IHC) staining. The average ratio of c-Myc (+) cells in bone marrow myeloblasts in the combined cohort was 32.08% (<1% to >90%; Figure 3A,B). Furthermore, Myc gene and c-Myc protein expression significantly correlated in the prospective cohort (r = 0.495; *p* = 0.014; Figure 3C). However, this correlation did not translate into a meaningful difference in c-Myc protein expression between the groups with and without CR, although the ratio of c-Myc (+) myeloblasts was higher the group without than with CR (37.81 ± 25.13% vs. 29.04 ± 19.75%, *p* = 0.151; Figure 3D).

### Factors Associated With c-Myc Protein Expression

To identify potential factors that might interfere with the c-Myc expression in *de novo* AML, we compared the demographic and laboratory features among patients with (n = 19) and without (n = 56) high c-Myc-immunopositivity. Sex (*p* = 0.849), cytogenetic (*p* = 0.193), and molecular (*p* = 0.307 for FLT3 ITD mutation; *p* = 0.751 for NPM1 mutation) risks did not significantly differ between these groups. However, AML patients high c-Myc-immunopositivity were younger (41.58 ± 14.88 vs. 44.37 ± 51.29 years; *p* = 0.031) and had more leukocytosis (90.10 ± 61.26 vs. 44.37 ± 51.29 (10^3^/μL); *p* = 0.002) than those without high c-Myc-immunopositivity (Table 2). Notably, age, sex, leukocytes, cohort, high-risk cytogenetics, FLT3 ITD, and NPM1 mutations were not significantly associated with high c-Myc-immunopositivity in univariate and multivariate analyses, suggesting that c-Myc protein expression is independent of most clinical features of AML (Table 3).

**TABLE 2:** Comparison of characteristics between patients with and without high^a^ c-Myc-immunopositivity in combined cohort (n = 75).

Variable	High c-Myc (n = 19)	Without high c-Myc (n = 56)	P
Sex (n, %)			0.849^b^
Male	12 (63.16)	32 (57.14)	
Female	7 (36.84)	24 (42.86)	
Age, y (mean ± SD)	41.58 ± 14.88	49.84 ± 13.91	0.031^c^
Leukocytes, 10^3^/μL (mean ± SD)	90.10 ± 61.26	44.37 ± 51.29	0.002^c^
Cytogenetics (n, %)			0.193^b^
Favorable	2 (10.53)	6 (10.71)	
Intermediate	10 (52.63)	33 (58.93)	
Unfavorable	7 (36.84)	10 (17.86)	
Undetermined	0 (0)	7 (12.50)	
Molecular risk (n, %)			
FLT3 ITD mutation	5 (26.32)	7 (12.50)	0.307^b^
NPM1 mutation	5 (26.32)	10 (17.86)	0.751^b^
Undetermined	2 (10.53)	15 (26.79)	0.209^b^

SD: standard deviation.

^a^ > 40% of myeloblasts in bone marrow are c-Myc (+).

^b^ Chi-squared.

^c^ *t*-tests.

**TABLE 3:** Factors associated with high^a^ c-Myc-immunopositivity in the combined cohort (n = 75).

	Univariate analysis			Multivariate analysis		
	OR	95% CI	P	OR	95% CI	p
Age (y)	1.00	0.97–1.03	0.979	1.01	0.96–1.05	0.769
Sex (female vs. male)	0.50	0.18–1.37	0.179	0.86	0.24–3.13	0.818
Leukocytes at diagnosis, 10^3^/μL	1.00	1.00–1.01	0.442	1.00	1.00–1.01	0.851
Prospective vs. retrospective cohort	1.20	0.44–3.30	0.724	1.93	0.54–6.93	0.311
High-risk cytogenetics (yes vs. no)	2.78	0.95–8.11	0.062	3.16	0.74–13.59	0.122
FLT3 ITD mutation (yes vs. no)	1.14	0.30–4.43	0.847	1.46	0.30–7.15	0.642
NPM1 mutation (yes vs. no)	0.45	0.11–1.85	0.268	0.42	0.08–2.12	0.295

^a^ > 40% of myeloblasts in bone marrow are c-Myc (+). CI, confidence interval; OR, odds ratio.

### High-Risk Cytogenetics and c-Myc-Immunopositivity as Biomarkers for “7 + 3” Induction Response Prediction

We examined whether c-Myc could be a potential surrogate marker to predict an induction response of AML to “7 + 3”. Because the presence of high-risk cytogenetics was significantly associated with “7 + 3” induction failure in the present cohort (data not shown), we also compared the predictive value of “7 + 3” induction response between high-risk cytogenetics and high c-Myc-immunopositivity. The results showed that the sensitivity of high c-Myc-immunopositivity and high-risk cytogenetics was respectively, 38.46 and 56.52%, and the specificity was 81.63 and 91.30%, respectively. High-risk cytogenetics was more accurate than high c-Myc-immunopositivity (79.71 vs. 66.67%). Notably, the specificity of high c-Myc-immunopositivity combined with high-risk cytogenetics reached 95.92%, suggesting that c-Myc could facilitate cytogenetics to identify AML patients who will respond to “7 + 3” induction chemotherapy more precisely than cytogenetics alone (Table 4).

**TABLE 4:** Prediction of response to “7 + 3” induction based on high^a^ c-Myc-immunopositivity and high-risk cytogenetics.

		Total (n)	Non-CR (n)	CR (n)	Sensitivity (%)	Specificity (%)	PPV (%)	NPV (%)	Accuracy (%)
High c-Myc-immunopositivity	Yes	19	10	9	38.46	81.63	52.63	71.43	66.67
	No	56	16	40					
High-risk cytogenetics	Yes	17	13	4	56.52	91.30	76.47	80.77	79.71
	No	52	10	42					
High c-Myc-immunopositivity + high-risk cytogenetics	Yes	7	5	2	19.23	95.92	71.43	69.12	69.33
	No	68	21	47					

^a^ > 40% of myeloblasts in bone marrow are c-Myc (+). CR, complete remission; NPV, negative predictive value; PPV, positive predictive value.

## Discussion

We validated our previous finding of a higher Myc signature gene expression in patients with *de novo* AML who do not achieve CR under “7 + 3” induction therapy compared with those who achieve CR. The present findings also found higher Myc gene expression in patients without, than with CR. Furthermore, Myc gene expression positively correlated with ratios (%) of c-Myc (+) bone marrow myeloblasts. Therefore, the ratio (%) of c-Myc (+) bone marrow myeloblasts could be a feasible and timely approach with which to predict a chemoresponse of *de novo* AML to “7 + 3” induction. However, the ratio (%) of c-Myc (+) myeloblasts was not significantly higher in patients without, than with CR. Nonetheless, having >40% c-Myc (+) myeloblasts and high-risk cytogenetics could predict a response to “7 + 3” induction with 81.63 and 91.30% of specificity. Notably, adding high c-Myc-immunopositivity to the high-risk cytogenetics further increased the specificity to 95.92%. This result suggested that c-Myc expression could facilitate cytogenetics to more precisely identify AML patients who are likely to respond to “7 + 3” induction chemotherapy.

Various potential mechanisms of Myc-related chemoresistance in solid cancers have been suggested. Chemoresistance associated with TCRP1- can be transcriptionally regulated by c-Myc in tongue and lung cancers (Jia et al., 2017). In addition, Myc and MCL1 might cooperatively promote chemotherapy-resistant breast cancer stem cells by regulating mitochondrial oxidative phosphorylation (Lee et al., 2017). Moreover, the c-Myc/miR-27b-3p/ATG10 axis regulates chemoresistance in colorectal cancer (Sun et al., 2020). Among hematological malignancies, crosstalk between Myc and p53 proteins might result in an inferior outcome of B-cell lymphomas (Yu et al., 2019). Increased Myc copy numbers comprise a negative prognostic factor for diffuse large B cell lymphoma (Schieppati et al., 2020). The expression of NKG2D ligands is regulated by c-Myc in AML and AML cell lines rendered resistant to cytarabine express more of the NKG2D ligands ULBP1/2/3 (Nanbakhsh et al., 2014). Consequently, NKG2DL upregulation rendered the cell lines more sensitive to NK cell-mediated lysis.

Based on the possible mechanisms responsible for c-Myc-associated chemoresistance in AML, determining whether c-Myc protein can be a feasible and timely clinical parameter to predict induction response remains a clinical challenge. A bone marrow content of ≥5% Myc (+) myeloblasts is an independent poor prognostic factor for AML with myelodysplasia-related changes (Yun et al., 2019). Moreover, Myc immunopositivity >6% is significantly associated with inferior overall, event-free, and relapse-free survival (Ohanian et al., 2019). These results suggest that Myc-immunopositivity is a critical prognostic factor in untreated AML, particularly in patients at higher risk for relapse. Although c-Myc-immunopositivity > 40% was not more accurate than high-risk cytogenetics in identifying “7 + 3” responders, the present findings indicated that c-Myc-immunopositivity could enhance predictive specificity when combined with high-risk cytogenetics. We selected >40% c-Myc (+) myeloblasts as the cutoff in the present study by testing the sensitivity and specificity according to different ratios (%) of c-Myc (+) myeloblasts. However, the optimal cutoff of c-Myc-immunopositivity still needs further validation. Notably, with a range from 0 to 100%, the average ratios of c-Myc-immunopositive myeloblasts were similar between the cohorts in the present study (32.08%) and that of Ohanian et al. (32%) (Ohanian et al., 2019), which indirectly validated our results.

The lack of a significant difference in c-Myc protein expression between the groups with and without CR remains unresolved. We did find a statistical correlation between Myc gene expression and the ratios (%) of c-Myc (+) myeloblasts (r = 0.495; *p* = 0.014). Ratios (%) of c-Myc (+) myeloblasts tended to be higher in the group without, than with CR (37.81 ± 25.13 vs. 29.04 ± 19.75), but the difference did not reach statistical significance. An insufficient number of patients might be the primary reason for this. The rapid degradation of Myc by the ubiquitin-proteasome system (Gregory and Hann, 2000) could be another explanation. In addition, many proteins are involved in the regulation of Myc protein stability and activity (Farrell and Sears, 2014), which might further interfere with c-Myc expression in myeloblasts in bone marrow specimens. The features of Myc have been further reflected in its potential clinical utilization. Direct c-Myc protein targeting does not seem to be an effective therapeutic approach. Conversely, targeting Myc transcription, disrupting Myc/Max dimerization, causing an interference in Myc protein stability, inhibiting Myc-associated cell cycle, and targeting metabolism through Myc target genes and cofactors are current anti-Myc strategies in cancer treatment (McAnulty and DiFeo, 2020).

The small patient cohort is a significant limitation of the present study. Because of this, we could not provide a validated model to predict a chemoresponse to “7 + 3” induction therapy. We are currently conducting a prospective observational study of more patients to develop a c-Myc protein-associated prediction model to overcome this clinical hurdle.

In summary, we showed that Myc and its related genes are responsible for chemoresistance in untreated *de novo* AML. The combination of cytogenetics and c-Myc-immunopositivity could be a feasible and timely approach with which to identify patients with *de novo* AML who are likely to achieve CR with “7 + 3” induction therapy. This therapy remains the standard of care for patients with *de novo* AML, especially for those without high c-Myc-immunopositivity and high-risk cytogenetics. However, other chemotherapeutic regimens (Fleischhack et al., 1998) or venetoclax-based induction (DiNardo et al., 2020) might be a solution for patients with high c-Myc-immunopositivity or high-risk cytogenetics. Prospective studies with more patients are needed to determine whether choosing different induction regimens based on this strategy can significantly improve the CR rates and overall survival among patients with recently diagnosed *de novo* AML.

## Data Availability

The datasets presented in this study can be found in online repositories. The names of the repository/repositories and accession number(s) can be found below: https://www.ncbi.nlm.nih.gov/geo/, GSE164894.

## References

[B1] Abdullah L. N. Chow E. K. (2013). Mechanisms of chemoresistance in cancer stem cells. Clin. Transl Med. 2 (1), 3. 10.1186/2001-1326-2-3 23369605 PMC3565873

[B2] Burnett A. Wetzler M. Löwenberg B. (2011). Therapeutic advances in acute myeloid leukemia. J Clin Oncol. 29 (5), 487–494. 10.1200/jco.2010.30.1820 21220605

[B3] Chen H. Liu H. Qing G. (2018). Targeting oncogenic Myc as a strategy for cancer treatment. Signal. Transduct Target. Ther. 3 (1), 5. 10.1038/s41392-018-0008-7 29527331 PMC5837124

[B4] Chiu Y. C. Hsiao T. H. Tsai J. R. Wang L. J. Ho T. C. Hsu S. L. (2019). Integrating resistance functions to predict response to induction chemotherapy in de novo acute myeloid leukemia. Eur. J. Haematol. 103 (4), 417–425. 10.1111/ejh.13301 31356696

[B5] De Kouchkovsky I. Abdul-Hay M. (2016). 'Acute myeloid leukemia: a comprehensive review and 2016 update'. Blood Cancer J. 6 (7), e441. 10.1038/bcj.2016.50 27367478 PMC5030376

[B6] Dinardo C. D. Jonas B. A. Pullarkat V. Thirman M. J. Garcia J. S. Wei A. H. (2020). Azacitidine and venetoclax in previously untreated acute myeloid leukemia. N. Engl. J. Med. 383, 617–629. 10.1056/nejmoa2012971 32786187

[B7] Elbadawy M. Usui T. Yamawaki H. Sasaki K. (2019). Emerging roles of C-Myc in cancer stem cell-related signaling and resistance to cancer chemotherapy: a potential therapeutic target against colorectal cancer. Int. J. Mol. Sci. 20 (9), 2340. 10.3390/ijms20092340 PMC6539579 31083525

[B8] Farrell A. S. Sears R. C. (2014). MYC degradation. Cold Spring Harb Perspect. Med. 4 (3), a014365. 10.1101/cshperspect.a014365 24591536 PMC3935390

[B9] Fleischhack G. Hasan C. Graf N. Mann G. Bode U. (1998). IDA-FLAG (idarubicin, fludarabine, cytarabine, G-CSF), an effective remission-induction therapy for poor-prognosis AML of childhood prior to allogeneic or autologous bone marrow transplantation: experiences of a phase II trial. Br. J. Haematol. 102 (3), 647–655. 10.1046/j.1365-2141.1998.00836.x 9722289

[B10] Gregory M. A. Hann S. R. (2000). c-Myc proteolysis by the ubiquitin-proteasome pathway: stabilization of c-Myc in Burkitt's lymphoma cells. Mol. Cel. Biol. 20 (7), 2423–2435. 10.1128/mcb.20.7.2423-2435.2000 PMC85426 10713166

[B11] Ho T.-C. Becker M. W. (2013). Defining patient-specific risk in acute myeloid leukemia. J. Clin. Oncol. 31 (31), 3857–3859. 10.1200/jco.2013.51.4307 24062389

[B12] Jia X. Zhang Z. Luo K. Zheng G. Lu M. Song Y. (2017). TCRP1 transcriptionally regulated by c-Myc confers cancer chemoresistance in tongue and lung cancer. Sci. Rep. 7 (1), 3744. 10.1038/s41598-017-03763-0 28623290 PMC5473818

[B13] Kim D. Paggi J. M. Park C. Bennett C. Salzberg S. L. (2019). Graph-based genome alignment and genotyping with HISAT2 and HISAT-genotype. Nat. Biotechnol. 37 (8), 907–915. 10.1038/s41587-019-0201-4 31375807 PMC7605509

[B14] Lee K.-M. Giltnane J. M. Balko J. M. Schwarz L. J. Guerrero-Zotano A. L. Hutchinson K. E. (2017). MYC and MCL1 cooperatively promote chemotherapy-resistant breast cancer stem cells via regulation of mitochondrial oxidative phosphorylation. Cel Metab. 26 (4), 633–647.e7. 10.1016/j.cmet.2017.09.009 PMC5650077 28978427

[B15] Liao Y. Smyth G. K. Shi W. (2014). featureCounts: an efficient general purpose program for assigning sequence reads to genomic features. Bioinformatics 30 (7), 923–930. 10.1093/bioinformatics/btt656 24227677

[B16] Liberzon A. Subramanian A. Pinchback R. Thorvaldsdottir H. Tamayo P. Mesirov J. P. (2011). Molecular signatures database (MSigDB) 3.0. Bioinformatics 27 (12), 1739–1740. 10.1093/bioinformatics/btr260 21546393 PMC3106198

[B17] Love M. I. Huber W. Anders S. (2014). Moderated estimation of fold change and dispersion for RNA-seq data with DESeq2. Genome Biol. 15 (12), 550. 10.1186/s13059-014-0550-8 25516281 PMC4302049

[B18] Mcanulty J. Difeo A. (2020). The molecular 'myc-anisms' behind myc-driven tumorigenesis and the relevant myc-directed therapeutics. Int. J. Mol. Sci. 21 (24), 9486. 10.3390/ijms21249486 PMC7764474 33322239

[B19] Nanbakhsh A. Pochon C. Mallavialle A. Amsellem S. Bourhis J. H. Chouaib S. (2014). c-Myc regulates expression of NKG2D ligands ULBP1/2/3 in AML and modulates their susceptibility to NK-mediated lysis. Blood 123 (23), 3585–3595. 10.1182/blood-2013-11-536219 24677544 PMC4198341

[B20] Ng S. W. K. Mitchell A. Kennedy J. A. Chen W. C. Mcleod J. Ibrahimova N. (2016). A 17-gene stemness score for rapid determination of risk in acute leukaemia. Nature 540 (7633), 433–437. 10.1038/nature20598 27926740

[B21] Ohanian M. Rozovski U. Kanagal-Shamanna R. Abruzzo L. V. Loghavi S. Kadia T. (2019). MYC protein expression is an important prognostic factor in acute myeloid leukemia. Leuk. Lymphoma 60 (1), 37–48. 10.1080/10428194.2018.1464158 29741984 PMC6226369

[B22] Sansom O. J. Meniel V. S. Muncan V. Phesse T. J. Wilkins J. A. Reed K. R. (2007). Myc deletion rescues Apc deficiency in the small intestine. Nature 446 (7136), 676–679. 10.1038/nature05674 17377531

[B23] Schieppati F. Balzarini P. Fisogni S. Re A. Pagani C. Bianchetti N. (2020). An increase in MYC copy number has a progressive negative prognostic impact in patients with diffuse large B-cell and high-grade lymphoma, who may benefit from intensified treatment regimens. Haematologica 105 (5), 1369–1378. 10.3324/haematol.2019.223891 31399522 PMC7193495

[B24] Schlosser I. Hölzel M. Hoffmann R. Burtscher H. Kohlhuber F. Schuhmacher M. (2005). Dissection of transcriptional programmes in response to serum and c-Myc in a human B-cell line. Oncogene 24 (3), 520–524. 10.1038/sj.onc.1208198 15516975

[B25] Sun W. Li J. Zhou L. Han J. Liu R. Zhang H. (2020). The c-Myc/miR-27b-3p/ATG10 regulatory axis regulates chemoresistance in colorectal cancer. Theranostics 10 (5), 1981–1996. 10.7150/thno.37621 32104496 PMC7019154

[B26] Vardiman J. W. Thiele J. Arber D. A. Brunning R. D. Borowitz M. J. Porwit A. (2009). The 2008 revision of the World Health Organization (WHO) classification of myeloid neoplasms and acute leukemia: rationale and important changes. Blood 114 (5), 937–951. 10.1182/blood-2009-03-209262 19357394

[B27] Vidal S. J. Rodriguez-Bravo V. Galsky M. Cordon-Cardo C. Domingo-Domenech J. (2014). Targeting cancer stem cells to suppress acquired chemotherapy resistance. Oncogene 33 (36), 4451–4463. 10.1038/onc.2013.411 24096485

[B28] Walter R. B. Othus M. Burnett A. K. Löwenberg B. Kantarjian H. M. Ossenkoppele G. J. (2015). Resistance prediction in AML: analysis of 4601 patients from MRC/NCRI, HOVON/SAKK, SWOG and MD anderson cancer center. Leukemia 29 (2), 312–320. 10.1038/leu.2014.242 25113226 PMC4318722

[B29] Yu L. Yu T.-T. Young K. H. (2019). Cross-talk between Myc and p53 in B-cell lymphomas. Chronic Dis. Translational Med. 5 (3), 139–154. 10.1016/j.cdtm.2019.08.001 PMC6926120 31891126

[B30] Yun S. Sharma R. Chan O. Vincelette N. D. Sallman D. A. Sweet K. (2019). Prognostic significance of MYC oncoprotein expression on survival outcome in patients with acute myeloid leukemia with myelodysplasia related changes (AML-MRC). Leuk. Res. 84, 106194. 10.1016/j.leukres.2019.106194 31357093 PMC7375354

