# Supplementary material for: Chemoresponse of de novo Acute Myeloid Leukemia to “7+3” Induction can Be Predicted by c-Myc-facilitated Cytogenetics
Source: Front Pharmacol. 2021 Apr 8;12:649267. doi: 10.3389/fphar.2021.649267 (PMC8061304; doi:10.3389/fphar.2021.649267)
Supplement: Supplementary file 1 [file table1.docx]

| **Supplemental table 1. Sensitivity and specificity of each cutoff of high c-Myc-immunopositivity** | | |
| --- | --- | --- |
| **Criterion** | **Sensitivity (%)** | **Specificity (%)** |
| > 0.01 | 96.15 | 6.12 |
| > 0.02 | 92.31 | 6.12 |
| > 0.05 | 84.62 | 18.37 |
| > 0.1 | 80.77 | 28.57 |
| > 0.2 | 65.38 | 44.90 |
| > 0.3 | 50.00 | 61.22 |
| > 0.4 | 38.46 | 81.63 |
| > 0.5 | 26.92 | 87.76 |
| > 0.6 | 11.54 | 93.88 |
| > 0.7 | 11.54 | 100.00 |
| > 0.9 | 0.00 | 100.00 |
