# Supplementary material for: Chemoresponse of de novo Acute Myeloid Leukemia to “7+3” Induction can Be Predicted by c-Myc-facilitated Cytogenetics
Source: Front Pharmacol. 2021 Apr 8;12:649267. doi: 10.3389/fphar.2021.649267 (PMC8061304; doi:10.3389/fphar.2021.649267)
Supplement: Supplementary file 2 [file table2.docx]

| **Supplemental Table 2. Comparisons of patients’ characteristics between the retrospective and prospective cohorts** | | | | |
| --- | --- | --- | --- | --- |
| **Variable** | **All patients**  **(n = 75)** | **Retrospective cohort**  **(n = 51)** | **Prospective cohort**  **(n = 24)** | **p-value** |
| **Sex (n, %)** |  |  |  | 0.209^a^ |
| Male | 44 (58.67) | 27 (52.94) | 17 (70.83) |  |
| Female | 31 (41.33) | 24 (47.06) | 7 (29.17) |  |
| **Age, years**  **(mean ± SD)** | 47.75 ± 14.51 | 45.86 ± 14.69 | 51.75 ± 13.57 | 0.102^b^ |
| **Leukocytes, 10^3^/μL**  **(mean ± SD)** | 56.34 ± 57.18 | 58.59 ± 60.08 | 51.35 ± 51.08 | 0.618^b^ |
| **Cytogenetics (n, %)** |  |  |  | 0.254^a^ |
| Favorable | 8 (10.67) | 5 (9.80) | 3 (12.50) |  |
| Intermediate | 43 (57.33) | 29 (56.86) | 14 (58.33) |  |
| Unfavorable | 17 (23.94) | 10 (19.61) | 7 (29.17) |  |
| Undetermined | 7 (9.33) | 7 (13.73) | 0 (0%) |  |
| **Molecular risk (n, %)** |  |  |  |  |
| FLT3 ITD mutation | 12 (16.00) | 8 (15.69) | 4 (16.67) | 0.744^a^ |
| NPM1 mutation | 15 (20.00) | 10 (19.61) | 5 (20.83) | 0.547^a^ |
| Undetermined | 17 (22.67) | 17 (33.33) | 0 (0%) | 0.003^a^ |
| SD, standard deviation  p-values determined using ^a^Chi-squared test and ^b^*t*-test | | | | |
